# DNA mismatch repair and CD133-marked cancer stem cells in colorectal carcinoma

**DOI:** 10.7717/peerj.5530

**Published:** 2018-09-11

**Authors:** Phaik-Leng Cheah, Jing Li, Lai-Meng Looi, Kean-Hooi Teoh, Diana Bee-Lan Ong, Mark J. Arends

**Affiliations:** 1 Division of Anatomical Pathology, Department of Pathology, Faculty of Medicine, University of Malaya, Kuala Lumpur, Malaysia; 2 Division of Pathology, Edinburgh Cancer Research Centre, University of Edinburgh, Edinburgh, United Kingdom

**Keywords:** Tumor side, Colorectal carcinoma, CD133, DNA mismatch repair protein, Cancer stem cells

## Abstract

**Background:**

Except for a few studies with contradictory observations, information is lacking on the possibility of association between DNA mismatch repair (MMR) status and the presence of cancer stem cells in colorectal carcinoma (CRC), two important aspects in colorectal carcinogenesis.

**Methods:**

Eighty (40 right-sided and 40 left-sided) formalin-fixed, paraffin-embedded primary CRC were immunohistochemically studied for CD133, a putative CRC stem cell marker, and MMR proteins MLH1, MSH2, MSH6 and PMS2. CD133 expression was semi-quantitated for proportion of tumor immunopositivity on a scale of 0–5 and staining intensity on a scale of 0–3 with a final score (units) being the product of proportion and intensity of tumor staining. The tumor was considered immunopositive only when the tumor demonstrated moderate to strong intensity of CD133 staining (a decision made after analysis of CD133 expression in normal colon). Deficient MMR (dMMR) was interpreted as unequivocal loss of tumor nuclear staining for any MMR protein despite immunoreactivity in the internal positive controls.

**Results:**

CD133 was expressed in 36 (90.0%) left-sided and 28 (70.0%) right-sided tumors (*p* < 0.05) and CD133 score was significantly higher in left- (mean ± SD = 9.6 ± 5.3 units) compared with right-sided tumors (mean ± SD = 6.8 ± 5.6 units) *p* < 0.05). dMMR was noted in 14 (35%) right-sided and no (0%) left-sided CRC. When stratified according to MMR status, dMMR cases showed a lower frequency of CD133 expression (42.9%) and CD133 score (mean ± SD = 2.5 ± 3.6 units) compared with pMMR tumors on the right (frequency = 84.6%; mean score ± SD = 9.2 ± 5.0 units) as well as pMMR tumors on the left (frequency = 90.0%; mean score ± SD = 9.6 ± 5.3 units) (*p* < 0.05). Interestingly, frequencies of CD133 immunoreactivity and CD133 scores did not differ between pMMR CRC on the right versus the left (*p* > 0.05).

**Conclusion:**

Proficient MMR correlated with high levels of CD133-marked putative cancer stem cells in both right- and left-sided tumors, whereas significantly lower levels of CD133-marked putative cancer stem cells were associated with deficient MMR status in colorectal carcinomas found on the right.

## Introduction

Colorectal carcinoma (CRC) is a leading cause of death worldwide and the most common malignancy in Malaysian males and the third most common in Malaysian females (Lim, Ganesanathan & Palaiyan, 2008). While there have been major advances in understanding the pathogenesis of this important tumor since the CRC tumorigenic model was proposed by Fearon & Vogelstein (1990), much still remains unclear. In recent years, interest has been mounting over the cancer stem cell hypothesis of carcinogenesis in which cancer stem cells are viewed as distinct cells within a tumor that possess the capacity to self-perpetuate, hence maintaining the immortality of the tumor and eventually giving rise to the heterogeneous lineages of the cancer (Clarke et al., 2006; Lopez et al., 2012). The cancer stem cell theory would implicate cancer stem cells as central to most tumor development, including CRC, and it would therefore be important to evaluate cancer stem cells and factors that may influence their function and roles to provide insight into this potentially important area.

The identification of the microsatellite instability (MSI) pathway, due to deficient DNA mismatch repair (dMMR) has been incriminated as an alternative in the development of CRC in families with Lynch syndrome (accounting for 2–4% CRC) and in ∼15% of sporadic cancers (Aaltonen et al., 1993; Ionov et al., 1993; Thibodeau, Bren & Schaid, 1993; Szylberg et al., 2015; Thompson & Spurdle, 2015). Deficient MMR arises due to failure of the DNA mismatch repair system that results in “instability” of microsatellites; microsatellites being tandem repeats of 1–6 nucleotides scattered in coding and non-coding regions, and the microsatellites together make up 3% of the genome (Hearne, Ghosh & Todd, 1992; Parsons et al., 1993; Subramanian et al., 2003). Insertions or deletions of the microsatellites can result in a “mutator” phenotype, affecting genes eg PTEN, BAX, TGFBRII, EGFR etc, that have critical functions in cell signaling, apoptosis and proliferation (Iacopetta, Grieu & Amanuel, 2010).

CD133 identified by the AC133 antigenic epitope, is the human homologue of murine Prominin-1, and is one of the most touted markers for the putative cancer stem cells in CRC (Lapidot et al., 1994; Bonnet & Dick, 1997). Identifying DNA mismatch repair (MMR) status and correlating this with CD133 expression could provide important information regarding the relationship between defective MMR and colorectal cancer stem cell function. Although some work has been carried out in this area, information remains limited and results are somewhat contradictory, with Park et al. (2014) and Neumann et al. (2012) demonstrating microsatellite stability with its implicit accompanying proficient DNA mismatch repair system in CRC being associated with high CD133, and Huh et al. (2010) the reverse. As deficient MMR is generally reported to occur more commonly in right-sided CRC (Yearsley et al., 2006; Iacopetta, Grieu & Amanuel, 2010; Benedix et al., 2012; Koi, Tseng-Rogenski & Carethers, 2018), the study was also designed to stratify the CRC cases according to location of the tumor, to determine if CD133 expression is associated with tumor side.

## Material and Methods

Colorectal carcinomas were categorized based on anatomical site viz those proximal to the splenic flexure as right-sided and those at the splenic flexure as well as distal to it as left-sided. Forty consecutive right-sided and 40 left-sided primary CRC, diagnosed between 1st January 2005 and 31st December 2007, were retrieved from the archives of the Department of Pathology, Faculty of Medicine, University of Malaya. All cases were histologically reviewed and only re-confirmed cases were entered into the study. Relevant clinical information was obtained from the surgical pathology examination request forms. The study was approved by the Institutional Review Board (IRB) of the University of Malaya Medical Centre (MEC 794.75) and carried out in compliance with the Declaration of Helsinki. Informed, written consent to undergo resection of the colorectal cancer was obtained from all patients. The Institutional Review Board of the University of Malaya Medical Centre accepts that additional consent from patients is not required for retrospective studies on archived material and which are observational in nature. Confidentiality of patients’ personal information was maintained by restricting access of data to the authors of the present work. Five 4 um sections were cut from the formalin-fixed, paraffin-embedded primary tumor tissue block selected during histopathological review and placed on platinum coated slides (Matsunami Glass Industries, Kishiwada, Japan) for subsequent immunohistochemical staining.

### CD133

Immunohistochemical staining with a rabbit polyclonal antibody to CD133 (1:200; Abcam ab19898, Cambridge, United Kingdom) and detection by the ultraView universal DAB detection kit (Ventana Medical Systems Inc., Tucson, AZ, USA) was carried out on the Ventana Benchmark XT autostainer (Ventana Medical Systems Inc., Tucson, AZ, USA). A case of glioblastoma, previously shown to be immunopositive for CD133 was used as a positive control and run with each batch. CD133 immunostaining was looked for at the glandular luminal surface (Ong et al., 2010). Expression was semi-quantitated for (1) proportion of CD133 immunopositivity in the CRC as 0 (negative), 1 (<5% tumor staining for CD133), 2 (6–25% staining), 3 (26–50% staining), 4 (51–75% staining) and 5 (>75% staining) and (2) intensity of staining 0 (negative), 1 (weak), 2 (moderate) and 3 (strong) (Chew, Teoh & Cheah, 2012). The final CD133 immunopositivity score was calculated as proportion of CD133 immunopositivity multiplied by intensity of staining and arbitrarily measured as “units”. The determination of the cut-off immunostaining positivity would be determined after comparing the CD133 immunostaining in CRC with that of the normal colonic mucosa, in those cases where the latter was available on the same tissue section as the CRC. After determination of the cut-off, the scores would be subcategorized as negative (0 units), low (1–5 units), intermediate (6–10 units) and high (11–15 units).

### DNA mismatch repair protein

Based on the understanding that the expression of DNA MMR gene products, MLH1, MSH2, MSH6 and PMS2, are known to fairly reflect MSI status (Pinol et al., 2005; Niessen et al., 2006; Yuan et al., 2015), immunohistochemical staining of the CRC cases was carried out for DNA MMR on the Ventana Benchmark XT autostainer (Ventana Medical Systems Inc., Tucson, AZ, USA) using monoclonal antibodies to MLH1 (1:100; BD Pharmingen, clone G168-728), MSH2 (1:800; BD Pharmingen, clone G219-1129), MSH6 (1:500; BD Transduction Laboratories, clone 44/MSH6) and PMS2 (1:100; BD Pharmingen, clone A16-4). Detection was via the ultraView universal DAB detection kit (Ventana Medical Systems Inc., Tucson, AZ, USA). Proficient MMR (pMMR) protein expression was defined as unequivocal tumor nuclear immunostaining in the presence of immunopositivity of the internal positive controls (lymphocytes, fibroblasts or normal enterocytes in the vicinity of the tumor). The tumor was classified as having deficient MMR (dMMR) when there was unequivocal loss of tumor nuclear staining for one or more of the MMR proteins in the presence of immunoreactivity in the internal positive controls. The case would be withdrawn whenever the internal controls failed.

Statistical analysis was performed using SPSS (IBM SPSS Statistics version 22; Armonk, NY, USA). For this study, categorical variables would be tested by Chi-square or Fisher’s exact test while the Independent Samples T-test or One-way Anova would be used for parametric, and Mann–Whitney U or Kruskal–Wallis for non-parametric continuous variables. Statistical significance was set as *p* < 0.05.

## Results

**Figure 1:**
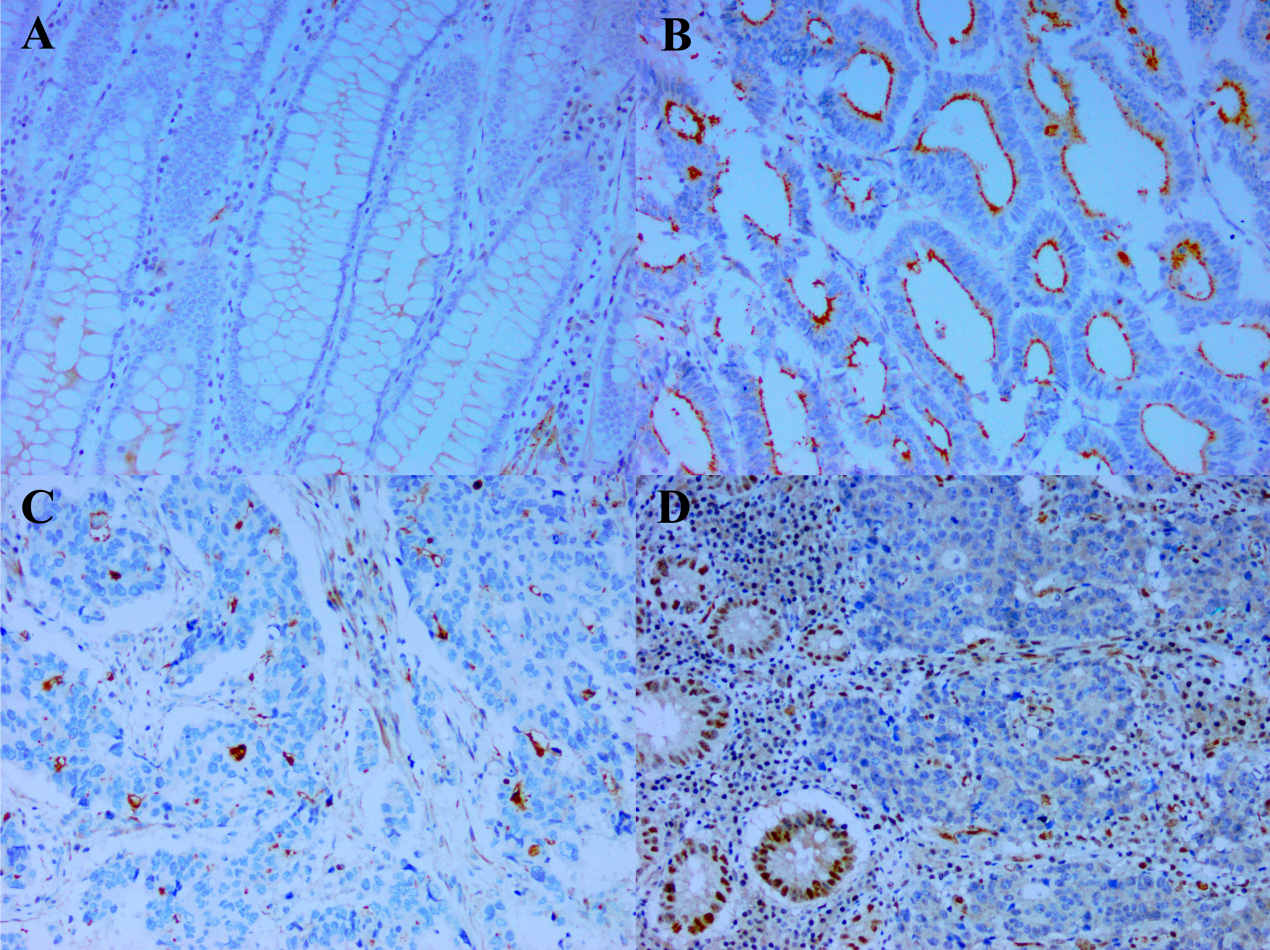
CD133 expression and deficient DNA mismatch repair. (A) The weak CD133 staining seen at the luminal border of some normal colonic crypts is considered equivocal and interpreted as negative (x200). In contrast, (B) is a case of colorectal carcinoma with strong CD133 staining of the glandular luminal surface (x200) and (C) a case of poorly differentiated colorectal carcinoma demonstrating CD133 staining as tiny cytoplasmic blobs which represent abortive glands (x200). (D) Colorectal carcinoma with deficient DNA mismatch repair (represented by loss of MLH1). Note the lack of immunoexpression of MLH1 in the colorectal carcinoma nuclei in the presence of immunopositivity in the internal positive controls (stromal cells, lymphocytes and nearby normal enterocytes) (x200)

The demographic data and clinical characteristics of the CRC entered into this study are shown in Table 1. Except for a slight preponderance of older patients (*p* = 0.043) and poorly differentiated tumors on the right (*p* = 0.025), there was no significant difference noted in gender and ethnicity of the patients, or size of tumor and TNM staging between the right- and left-sided tumors. Seventy-four of the CRC had some adjacent normal colonic mucosa. Of these, 30 (40.5%) demonstrated weak CD133 staining in the luminal borders of the normal colonic crypts (Fig. 1A), coupled occasionally with membranous staining of the crypt cells. No staining was observed in 44 of the cases. When staining was present, it was observed in few to majority (>75%) of the normal colonic crypts. Based on these observations, it was decided that weak staining intensity in whatever percentage of the tumor would be considered equivocal and interpreted as negative. Cases without CD133 staining were similarly negative. The CD133 score of the case in either of the above situations would then be calculated to be zero (0) units. Therefore, only staining of at least moderate intensity would be used in the calculation of the score i.e., proportion of CD133 immunopositivity multiplied by intensity of staining. Although when expressed, CD133 decorated the luminal surface of the tumor glands when the glandular structures could be clearly deciphered (Fig. 1B), tumors with poor differentiation commonly demonstrated CD133 as tiny blobs which probably represent abortive glands, in the tumor cellular cytoplasm (Fig. 1C) (Mia-Jan et al., 2013). Table 2 shows the expression of CD133 and MMR status as per size, stage and differentiation of the CRC. CD133 was expressed in 80.0% (64/80) of the CRC. CD133 scores of the cases ranged between 0–15 units with mean ± SD = 8.2 ± 5.6 units. The size, stage and differentiation of the CRCs were not linked to CD133 expression in terms of frequency of immunopositivity and CD133 score. For the MMR status, dMMR (Fig. 1D) was noted in 14/80 (17.5%) of the cases, while a majority (82.5%) of cases were pMMR. Of the dMMR tumors, 10 had concomitant loss of MLH1 and PMS2, two concomitant MSH2 and MSH6 loss and one each of isolated loss of either PMS2 or MSH6. dMMR tumors tended to be larger and less differentiated compared with pMMR ones.

**Table 1:** Demographic data and clinical characteristics. Comparison between cases of right-sided and left-sided colorectal carcinoma.

**Case characteristics**		**Right-sided** (*n* = 40)	**Left-sided** (*n* = 40)	*p*
Age (yrs)	Mean	64.6	60.2	0.043*
	Range	15–87	32–81	
Sex	Male	18 (45.0%)	26 (65.0%)	0.072
	Female	22 (55.0%)	14 (35.0%)	
	M:F	1.0:1.2	1.0:0.5	
Race	Chinese	25 (62.5%)	17 (42.5%)	0.089
	Indian	9 (22.5%)	8 (20.0%)	
	Malay	4 (10.0%)	13 (32.5%)	
	Others	2 (5.0%)	2 (5.0%)	
Tumor size	0–5 cm	18 (45.0%)	21 (52.5%)	0.493
	6–10 cm	20 (50.0%)	19 (47.5%)	
	11–15 cm	2 (5.0%)	0 (0%)	
TNM stage	I	4 (10.0%)	3 (7.5%)	0.900
	II	14 (35.0%)	13 (32.5%)	
	III	20 (50.0%)	20 (50.0%)	
	IV	2 (5.0%)	4 (10.0%)	
Differentiation	Well	1 (2.5%)	2 (5.0%)	0.025*
	Moderate	27 (67.5%)	35 (87.5%)	
	Poor	12 (30.0%)	3 (7.5%)	

**Table 2:** CD133 expression and DNA mismatch repair (MMR) status. The CD133 expression and MMR status as per size, TNM stage and differentiation of the colorectal carcinoma (*n* = 80).

**Colorectal carcinoma characteristics**		**CD133**										**MMR status**		
		**Immunoexpression**			**Score**							**pMMR***n* (%)	dMMR *n* (%)	
		Pos *n* (%)	Neg *n* (%)	*p*	Units	*p*	Neg	Low *n* (%)	Intermediate *n* (%)	High *n* (%)	*p*			*p*
Size (cm)	0–5	35 (54.7)	4 (25.0)	0.071	9.5 ± 4.8	0.084	4 (25.0)	3 (37.5)	15 (60.0)	17 (54.8)	0.087	36 (54.5)	3 (21.4)	0.004*
	6–10	28 (43.8)	11 (68.8)		7.3 ± 6.0		11 (68.8)	4 (50.0)	10 40.0)	14 (45.2)		30 (45.5)	9 (64.3)	
	11–15	1 (1.6)	1 (6.3)		2.0 ± 2.8		1 (6.3)	1 (12.5)	0 (0)	0 (0)		0 (0)	2 (14.3)	
TNM Stage	I	7 (10.9)	0 (0)	0.250	11.4 ± 3.2	0.356	0 (0)	0 (0)	3 (12.0)	4 (12.9)	0.353	6 (9.1)	1 (7.1)	0.889
	II	19 (29.7)	8 (50.0)		7.2 ± 6.1		8 (50.0)	3 (37.5)	7 (28.0)	9 (29.0)		22 (33.3)	5 (35.7)	
	III	32 (50.0)	8 (50.0)		8.5 ± 5.6		8 (50.0)	3 (37.5)	12 (48.0)	17 (54.8)		32 (48.5)	8 (57.1)	
	IV	6 (9.4)	0 (0)		7.5 ± 3.8		0 (0)	2 (25.0)	3 (12.0)	1 (3.2)		6 (9.1)	0 (0)	
Differentiation	Well	3 (4.7)	0 (0)	0.353	9.3 ± 3.1	0.123	0 (0)	0 (0)	2 (8.0)	1 (3.2)	0.078	3 (4.5)	0 (0)	0.008*
	Mod	51 (79.7)	11 (68.8)		8.8 ± 5.5		11 (68.8)	4 (50.0)	20 (80.0)	27 (87.1)		55 (83.3)	7 (50.0)	
	Poor	10 (15.6)	5 (31.3)		5.5 ± 5.6		5 (31.3)	4 (50.0)	3 (12.0)	3 (9.7)		8 (12.1)	7 (50.0)	

**Notes.**

n number pMMR proficient DNA mismatch repair dMMR deficient DNA mismatch repair pos positive neg negative mod moderate

When the cases were stratified according to tumor location and MMR status (Table 3), CD133 immunopositivity was identified in 28 of the 40 (70.0%) right-sided and 36 of the 40 (90.0%) left-sided CRC (*p* = 0.025). CD133 scores ranged between 0–15 units on both sides, with right-sided scores (mean ± SD = 6.8 ± 5.6 units) being lower than left (mean ± SD = 9.6 ± 5.3 units) (*p* = 0.021). Taking MMR status into account, only 6 of the 14 dMMR CRC (42.9%) were CD133 immunopositive in comparison with 58 of the 66 pMMR cases (87.9%) (*p* = 0.0007). CD133 scores of dMMR cases (range = 0–10 units; mean ± SD =2.5 ± 3.6 units) were also lower (*p* = 0.00004) than pMMR CRC (range = 0–15 units; mean ± SD = 9.4 ± 5.1 units). Frequency of CD133 immunopositivity did not differ (*p* = 0.702) between pMMR tumors on the right (84.6%; 22/26) and the left (90.0%; 36/40). Similarly, CD133 scores of the right-sided pMMR CRC (range = 0–15; mean ± SD = 9.2 ± 5.0 units) did not differ (*p* = 0.625) from those on the left (range = 0–15; mean ± SD = 9.6 ± 5.3 units). When CD133 scores were subcategorized to negative, low, intermediate and high, the frequency of dMMR cases in the higher score category was lower than pMMR cases both on the right (*p* = 0.0002) and left (*p* = 0.00006). Interestingly, there was a marginal increase of left-sided pMMR CRCs in the higher score category compared with pMMR cases on the right (*p* = 0.034).

**Table 3:** DNA mismatch repair protein (MMR) status and CD133 expression. CD133 immunoexpression and score being stratified by MMR status in right-sided and left-sided colorectal carcinomas.

**Right-sided colorectal carcinoma**							**Left-sided colorectal carcinoma**						
	**CD133**							**CD133**					
	**Immuno-expression**		**Score**					**Immuno-expression**		**Score**		
	**Positive**	**Negative**	**Units**	**Low***n* (%)	**Intermed***n* (%)	**High***n* (%)		**Positive**	**Negative**	**Units**	**Low***n* (%)	**Intermed***n* (%)	**High***n* (%)
dMMR	6 (21.4)	8 (66.7)	2.5 ± 3.6	3 (100.0)	3 (18.8)	0 (0)	dMMR	0 (0)	0 (0)	0 (0)	0 (0)	0 (0)	0 (0)
pMMR	22 (78.6)	4 (33.3)	9.2 ± 5.0	0 (0)	13 (81.3)	9 (100.0)	pMMR	36 (100.0)	4 (100.0)	9.6 ± 5.3	5 (100.0)	9 (100.0)	22 (100.0)

**Notes.**

n number NA not applicable intermed intermediate dMMR deficient DNA mismatch repair pMMR proficient DNA mismatch repair

## Discussion

Except for a slight preponderance of older patients and poorly differentiated tumors on the right, the right- and left-sided CRCs showed neither difference in patients’ gender and ethnicity, nor in size and stage of the tumors, prompting the notion that both the right- and left-sided CRCs in this study were reasonably matched. Immunopositivity for CD133 was noted in 80.0% (64/80) of the CRC in this study, which falls into the range previously described in other studies (Yang et al., 2011; Del Rio et al., 2013). The expression was not associated with size, stage or differentiation of the CRC, as was also noted by Hong et al. (2015). For DNA mismatch repair status, 66 (82.5%) cases were pMMR. In comparison, dMMR was detected in 17.5% of our cases, at a rate comparable to the 6 to 20% reported in other populations (Kim & Kang, 2014). Of the dMMR tumors, 10 had concomitant loss of MLH1 and PMS2, 2 concomitant MSH2 and MSH6 loss and one each of isolated loss of either PMS2 or MSH6. As a further point, in the interpretation of MMR findings, it is necessary to consider the dictates of the obligatory partnerships of eukaryotic MMR proteins (Sinicrope & Sargent, 2012). In the instances of concomitant loss of MLH1 and PMS2 in 10 cases and concurrent loss of MSH2 and MSH6 in two cases, the loss of PMS2 and MSH6 are most likely secondary events consequent upon loss of their respective obligatory binding partners i.e., MLH1 and MSH2. On the contrary, the isolated MSH6 loss in one CRC and loss of PMS2 in another appear to be independent events without loss of their binding partners. The tendency for dMMR tumors to be larger and less differentiated as noted here has also been observed and reported (Iacopetta, Grieu & Amanuel, 2010; Batur et al., 2016).

In this study, dMMR was observed exclusively in right-sided tumors. The predominance of dMMR on the right has been previously reported (Yearsley et al., 2006; Iacopetta, Grieu & Amanuel, 2010; Benedix et al., 2012) although as Cushman-Vokoun et al. (2013) cautions, studies on colorectal carcinoma selected for familial Lynch syndrome screening may show dMMR as high as 48% in left-sided tumors. Our population was unselected and it is not surprising that dMMR was not detected in any of the left-sided tumors. Interestingly, CD133 expression, was in terms of frequency of expression (70.0% right and 90.0% left) and scores (right: mean ± SD = 6.8 ± 5.6 units; left: mean ± SD = 9.6 ± 5.3 units on left) significantly lower in right-sided compared with left-sided tumors. Thus, pMMR and high level CD133 expression appear to be more prominent in left-sided tumors, indicating a possible association between CD133-marked putative cancer stem cells and proficient MMR. Although proper outcome assessment of pMMR and CD133-positive tumors would require larger study populations and longer follow-up, which was not included as part of this study, expression of CD133 (Bin et al., 2014; Kashihara et al., 2014) and proficient MMR or MSS (microsatellite stability) (Lin et al., 2014; Seppala et al., 2015) in CRC are generally associated with poorer outcomes. Our study results may therefore indirectly imply that left-sided tumors could be encumbered with two negative-prognostic impact markers. Notwithstanding the above, a large series has shown a bias for a better outcome in left-sided CRC (Loupakis et al., 2015; Price et al., 2015) making the argument that other factors, which have not been interrogated here, also influence the final outcome of cases of CRC.

Stratification of the tumors according to MMR status demonstrated a significantly lower frequency of CD133 immunoexpression as well CD133 score (42.9%; 2.5 ± 3.6 units) in dMMR or microsatellite instability-high (MSI-H) CRC compared with pMMR (MSS) cases, both on the right (84.6%; 9.2 ± 5.0 units) as well as the left (90.0%; 9.6 ± 5.3 units). At this juncture, it should be evident that the ratio of dMMR to pMMR cases will determine the final frequency and score of a cohort. Hence, the lower CD133 observed in the CRCs on the right compared with those on the left as discussed earlier must take this into cognizance. No difference was observed in the frequency of CD133 immunopositivity or CD133 scores between pMMR cases occurring on the right and left. The observation of a marginal increase of left-sided pMMR cases in the higher CD133 score category compared with right-sided pMMR cases is noteworthy. While the lack of difference in frequency of positivity and scores leads to the reasoning that CD133 is less likely to be associated with side but more likely with MMR status of the tumor, the observation that more left-sided pMMR cases appeared to be in the high CD133 score category will need larger studies to confirm whether the finding is spurious or has its implications. Nevertheless, this does not detract from the main conclusion that putative CD133-marked stem cells seem to be associated with proficient DNA mismatch repair.

The finding of lower CD133 in dMMR CRC in this study would appear to be more akin to those of Neumann et al.’s (2012) and Park et al.’s (2014) rather than that of Huh et al. (2010). Neumann et al. (2012) studied CD133 and MLH1 immunohistochemically in right-sided colonic cancers and noted that CD133 positivity and pMMR were both more common in cases with distant metastasis. Park et al. (2014) demonstrated that high CD133 immunopositivty was seen more frequently in MSS CRC. On the contrary, Huh et al. found that CD133 mRNA in their cases of CRC was significantly correlated with MSI-H and by inference dMMR. Notwithstanding the above, the authors also noted that only three out of five cases with CD133 positive immunohistochemical staining, as defined by higher CD133 expression in the cancer compared with the normal colon, showed similar higher CD133 mRNA compared with the normal. Sgambato et al. (2010) had also found that CD133 protein evaluated by flow cytometry did not correlate with its mRNA expression level. This then raises the question as to the whether CD133 mRNA should be assumed to equate with CD133 protein expression in CRC tissues. If not, this may explain Huh et al.’s (2010) observation that CD133 is associated with MSI-H, which is in contradistinction to the conclusions of Neumann et al. (2012), Park et al. (2014) and this study. Possible reasons for the association of CD133 with proficient DNA mismatch repair remain unclear. Suggestions of a link via epithelial-mesenchymal transition (EMT), whereby pMMR supports EMT and increased “stemness” of cells; or a situation when increased methylation of promoter CpG islands of CD133 and MLH1 may occur concurrently are possibilities to be considered (Park et al., 2014). Although there is little work done in this area, it is plausible that colorectal cancer stem cells may significantly benefit from a proficient DNA mismatch repair system with a stable microsatellite milieu that promotes continued viability and self-renewal characteristics. Whether the increased capacity for DNA repair noted in human pluripotent stem cells in a study by Luo et al. (2012) applies to the MMR system in CRC stem cells will need further investigation.

## Conclusions

This study which addresses the DNA mismatch repair status and colorectal cancer stem cells of 40 right- and 40 left-sided colorectal carcinomas showed that proficient MMR correlated with high levels of CD133-marked putative cancer stem cells in both right- and left-sided tumors, whereas significantly lower levels of CD133-marked putative cancer stem cells were associated with deficient MMR status in colorectal carcinomas found on the right.

##  Supplemental Information

10.7717/peerj.5530/supp-1 Supplemental Information 1 CD133 versus MMR status of colorectal carcinoma cases

## Additional Information and Declarations

### Data Availability

The following information was supplied regarding data availability: The raw data are provided in the Supplemental File.
