# Supplementary material for: DNA mismatch repair and CD133-marked cancer stem cells in colorectal carcinoma
Source: PeerJ. 2018 Sep 11;6:e5530. doi: 10.7717/peerj.5530 (PMC6138039; doi:10.7717/peerj.5530)
Supplement: Supplemental Information 1 [file peerj-06-5530-s001.pdf]

	Side	CD133	MMR
R1	L	0	
R2	L	6	
R3	L	15	
R4	L	4	
R5	L	12	
R6	L	12	
R7	L	15	
R8	L	6	
R9	L	2	
R10	L	15	
R11	L	12	
R12	L	0	
R13	L	10	
R14	L	12	
R15	L	15	
R16	L	6	
R17	L	9	
R18	L	12	
R19	L	12	
R20	L	15	
R21	L	15	
R22	L	15	
R23	L	15	
R24	L	15	
R25	L	4	
R26	L	6	
R27	L	12	
R28	L	12	
R29	L	12	
R30	L	15	
R31	L	2	
R32	L	9	
R33	L	0	
R34	L	4	
R35	L	6	
R36	L	0	
R37	L	8	
R38	L	15	
R39	L	15	
R40	L	15	
R41	R	10	
R42	R	0	dMMR
R43	R	15	dMMR
R44	R	0	
R45	R	4	
R46	R	0	dMMR
R47	R	4	dMMR

R48	R	10	dMMR
R49	R	15	
R50	R	8	
R51	R	6	
R52	R	0	
R53	R	12	dMMR
R54	R	15	
R55	R	9	
R56	R	10	
R57	R	15	
R58	R	10	
R59	R	10	
R60	R	6	
R61	R	0	
R62	R	9	
R63	R	15	
R64	R	15	
R65	R	2	
R66	R	6	dMMR
R67	R	0	
R68	R	0	dMMR
R69	R	6	dMMR
R70	R	12	dMMR
R71	R	0	
R72	R	0	dMMR
R73	R	6	
R74	R	9	
R75	R	10	
R76	R	0	
R77	R	15	dMMR
R78	R	0	
R79	R	0	
R80	R	9	dMMR
